# On the Detection of Population Heterogeneity in Causation Between Two Variables: Finite Mixture Modeling of Data Collected from Twin Pairs

**DOI:** 10.1007/s10519-024-10207-9

**Published:** 2024-11-26

**Authors:** Philip B. Vinh, Brad Verhulst, Hermine H. M. Maes, Conor V. Dolan, Michael C. Neale

**Affiliations:** 1 Department of Human and Molecular Genetics, Virginia Commonwealth University, Richmond, USA; 2 Virginia Institute for Psychiatric and Behavioral Genetics, Virginia Commonwealth University, Richmond, VA USA; 3 Department of Psychiatry and Behavioral Sciences, Texas A&M University, College Station, USA; 4 Department of Psychiatry, Virginia Commonwealth University, Richmond, USA; 5 Department of Biological Psychology, Vrije Universiteit, Amsterdam, Netherlands

**Keywords:** Causality, Mixture modeling, Twin design, Statistical modeling

## Abstract

Causal inference is inherently complex and relies on key assumptions that can be difficult to validate. One strong assumption is population homogeneity, which assumes that the causal direction remains consistent across individuals. However, there may be variation in causal directions across subpopulations, leading to potential heterogeneity. In psychiatry, for example, the co-occurrence of disorders such as depression and substance use disorder can arise from multiple sources, including shared genetic or environmental factors (common causes) or direct causal pathways between the disorders. A patient diagnosed with two disorders might have one recognized as primary and the other as secondary, suggesting the existence of different types of comorbidity. For example, in some individuals, depression might lead to substance use, while in others, substance use could lead to depression. We account for potential heterogeneity in causal direction by integrating the Direction of Causation (DoC) model for twin data with finite mixture modeling, which allows for the calculation of individual-level likelihoods for alternate causal directions. Through simulations, we demonstrate the effectiveness of using the Direction of Causation Twin Mixture (mixDoC) model to detect and model heterogeneity due to varying causal directions.

**Supplementary Information:**

The online version contains supplementary material available at 10.1007/s10519-024-10207-9.

## Introduction

Determining whether a risk factor has a causal relationship with a disease is essential for improving our understanding of disease mechanisms and informing further research. While randomized experiments, including randomized controlled trials, are widely used to establish causal relationships, they are often infeasible or unethical in many scenarios and cannot rule out reciprocal causal effects. In such situations, it is necessary to use quasi-experimental or correlational designs to illuminate causal processes (Shadish et al. 2002). However, drawing valid causal conclusions in non-experimental approaches is challenging because causal inference relies on strong assumptions that can be difficult to test.

For example, the high co-occurrence of depression and alcohol use disorder (AUD) (Grant & Harford 1995; Hasin et al. 2018) may arise from multiple distinct reasons. Some individuals with depression may use psychoactive substances to alleviate their symptoms (i.e., self-medication; Polimanti et al. 2019), while others who frequently drink excessively may develop symptoms of depression (Fergusson et al. 2009). Another possibility is that both disorders result from a shared pathophysiology (Zhou et al. 2017). These different explanations for the association between substance use and depression may coexist within a population, resulting in heterogeneous causal pathways. Our goal is to examine the feasibility of a research design and statistical method that can disentangle these complex heterogeneous causal relationships. To this end, we have adapted the Direction of Causation (DoC) model (Gillespie et al. 2003; Heath et al. 1993; Maes et al. 2021; McAdams et al. 2021; Neale & Cardon 1992; Verhulst & Estabrook 2012) with finite mixture modeling to allow for heterogeneity in the population.

### The Direction of Causation (DoC) twin model

The DoC model is a specific instance of the bivariate classical twin design (CTD). In the CTD monozygotic (MZ) and dizygotic (DZ) twins are measured on the same traits. Greater covariance of MZ pairs than DZ pairs is expected if genetic factors influence the trait, since members of an MZ twin pair have nominally identical genomes, whereas DZ twins share half of their alleles identically by descent.

The univariate twin model decomposes phenotypic variance into genetic and environmental sources, and the bivariate model similarly decomposes the phenotypic (2 × 2) covariance matrix of two phenotypes. This decomposition provides insight into the genetic and environmental contributions to phenotypic variance and covariance or comorbidity. Previous research has demonstrated that cross-sectional data collected from in the CTD can be used to test hypotheses concerning the causal relationship between two traits.

Differences in the heritability of bivariate twin data can be leveraged to investigate the direction of causality between the phenotypes. Given certain assumptions, the Direction of Causation (DoC) twin model can be used to test specific hypotheses concerning unidirectional or bidirectional causal relationships. The key information needed for this are the cross-twin cross-trait correlations (Duffy and Martin 1994; Heath et al. 1993; Maes et al. 2021; McAdams et al. 2021; Neale and Cardon 1992). For instance, if "X causes Y," the pattern of MZ and DZ cross-twin cross-trait covariances is predicted to be similar to the twin correlations for X (the causal trait). The cross-twin cross-trait covariance can arise from five potential sources in the DoC twin model. These sources are: (1) the additive genetic covariance (r_A_), representing the genetic contribution to the covariance between traits X and Y; (2) the shared environmental covariance (r_C_), which accounts for environmental factors shared between twins that influence both traits; (3) the unique environmental covariance (r_E_), which captures environmental influences unique to each twin that affect both traits; (4) the causal effect of trait X on trait Y, representing the direct influence of the first trait on the second; and (5) the causal effect of trait Y on trait X, representing the reverse direction of causality. These five sources may account for observed patterns of covariance in MZ and DZ twin pairs. However, due to model identification limitations, only three of these five parameters can be estimated simultaneously in the DoC model.

For any two traits with sufficiently different sources of phenotypic variance, the DoC model can be used to investigate the direction of causation. Suppose, for example, the variance of trait X consists of additive genetic (A) and unshared environment (E) components, while the variance of trait Y consists of shared (C) and unshared (E) environmental components. If X causes Y, the cross-twin, cross-trait covariance will reflect the heritable component in X and the within-person covariance between X and Y. By contrast, if Y causes X, the cross-twin, cross-trait covariance will mirror the common environment component in Y. The larger the difference in the underlying genetic and environmental variance components of the two traits, the easier it is to resolve the causal directions. In practice, both traits may be influenced by all three sources of variance, and as long as the proportions of variance differ and the sample size is sufficient, it is possible to resolve the direction of causation (Heath et al. 1993). Therefore, the DoC model provides a viable method for illuminating causal effects in cross-sectional twin data.

### Finite mixture modeling to understand causality in heterogeneous populations

Most causal modeling, including the DoC model, involves fitting alternative models to data under the assumption of homogeneity. This assumption implies that the putative causal model holds for all individuals in the population. The statistical counterpart of this assumption is that the observations are identically distributed (Brand and Thomas 2013; Xie 2013). Multiple causal processes within the same population imply causal heterogeneity, which almost certainly violates the identical distribution assumption. Failure to account for causal heterogeneity may give rise to parameter bias, and incorrect or inconsistent causal inferences (Muthén 1989).

The role of causality in comorbidity is difficult to ascertain because comorbidity may be due to shared risk factors (confounding), direct causal relationships, or both. In the case of causal heterogeneity, these are not mutually exclusive (Neale and Kendler 1995). Across psychiatric disorders, comorbidity is often the rule rather than the exception (Kessler et al. 1994; McGrath et al. 2020). If the association between comorbid disorders (e.g., depression and substance) is causal, but the direction of the causal relationship depends on latent class membership—that is, in one class, depression causes substance use, and in another, substance use causes depression—the data will follow a bivariate mixture distribution of two bivariate distributions. In this model, the parameter ω_i_ represents the proportion in which X causes Y, 1−ω_i_, represents the proportion of the population in which Y causes X. Modeling this type of causal heterogeneity can be done by means of finite mixture modeling (Dolan and van der Maas 1998; Lubke and Muthén, 2005; McLachlan and Peel 2000; Vermunt and Magidson 2014; Yung 1997).

### Integrating mixture modeling with the direction of causation model

Given that the DoC twin model offers the means to address the direction of causation, it seems plausible that a mixture distribution model may be able to detect population heterogeneity in this respect. Finite mixture modeling of twin data has been considered before. However, this was to conduct genetic covariance structure analysis in the absence of zygosity information (Benyamin et al. 2006; Neale 2003), or to investigate (latent) group differences in genetic and environmental variance components (Gillespie and Neale 2006). In the context of the DoC model, finite mixture modeling can be used to identify subgroups characterized by different causal directions (e.g., X causes Y vs. Y causes X), offering a more nuanced understanding of comorbidity. By combining the DoC model with finite mixture modeling, we aim to address the limitations of assuming causal homogeneity and explore the feasibility of detecting heterogeneity in causal directions within a population. This integrated approach offers a more comprehensive framework for understanding the complex relationships between traits, particularly in cases where causal pathways differ across subgroups.

The present study seeks to evaluate the feasibility of integrating finite mixture modeling with the DoC model to account for causal heterogeneity. We aim to: (i) determine whether the mixture model can accurately recover parameter values used in data simulation; (ii) compare the fit of mixture and non-mixture DoC models; and (iii) assess the posterior probabilities of individual classification into distinct causal subgroups. Ultimately, this research aims to provide insights into how heterogeneous causal relationships can be better understood using twin data.

## Materials and Methods

**Fig. 1:**
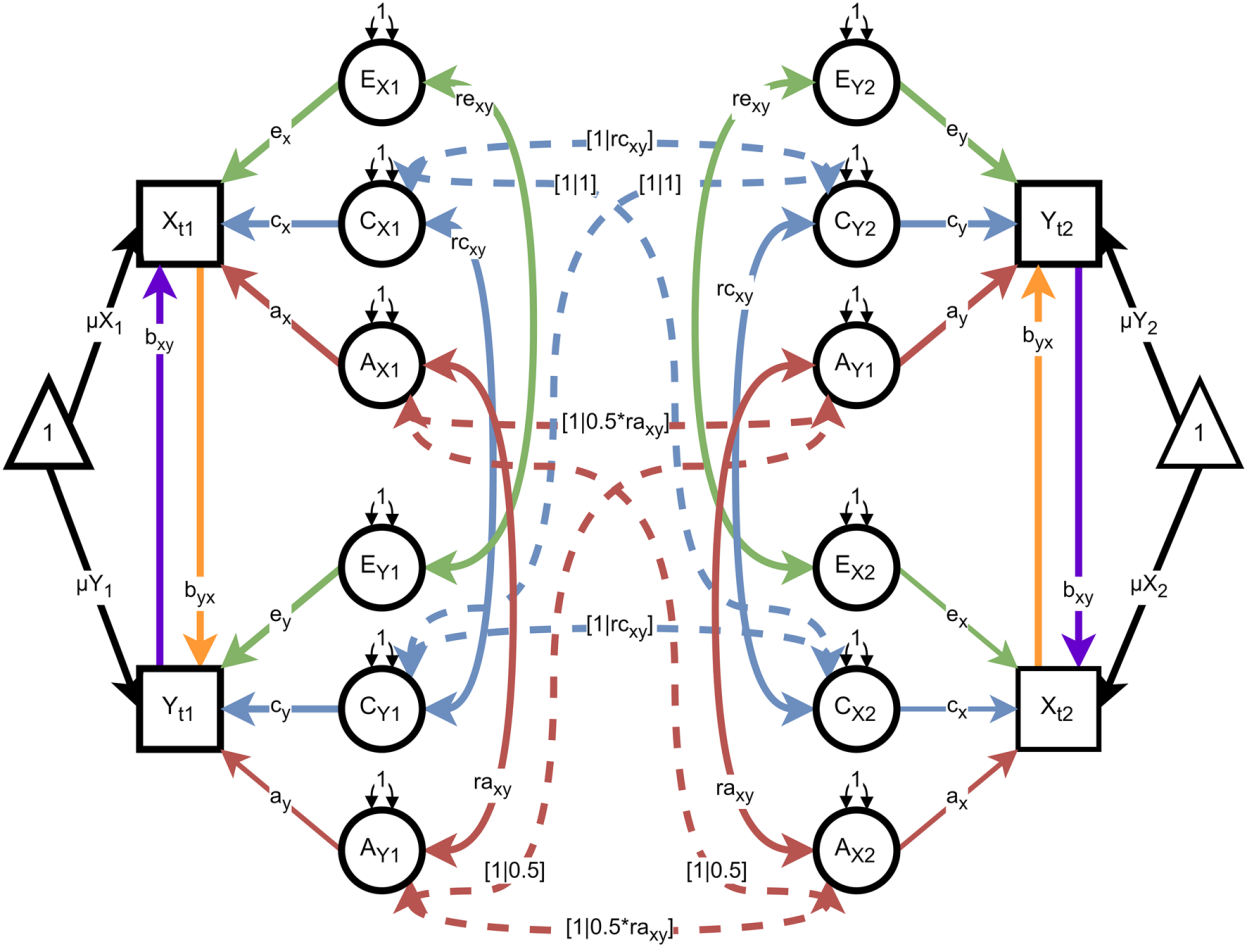
Path diagram representation of the Direction of Causation twin model. The model as shown is not identified. The diagram follows standard structural equation modeling notation, where circles represent latent variables, squares represent observed variables, double-headed arrows represent covariances/variances, and single-headed arrows represent regressions. The dashed lines are added for visual clarity to show the cross-twin covariances. The notation "[1|0.5*ra_xy_]" indicates that for monozygotic (MZ) twins, the genetic correlation between traits X and Y is fully shared (1), whereas for dizygotic (DZ) twins, the genetic correlation is half-shared (0.5). Other model parameters, such as a, c, e represent additive genetic (A), shared environmental (C), and unique environmental (E) influences, respectively

Figure 1 shows a path diagram of the DoC model, including all possible paths between two traits that can be estimated using cross-sectional twin data. In a bivariate ACE model, as depicted, each trait is influenced by latent additive genetic (*A*), shared environmental (*C*), and unique environmental (*E*) variance components. The traits may covary and these covariances are represented by correlations between the components (*r*_*A*_, *r*_*C*_, and *r*_*E*_*)*. Additionally, causal effects between X and Y are represented by the direct regression paths between the phenotypes (parameters b_xy_, b_yx_). Therefore, the five potential sources of covariance in the DoC model are: (1) the additive genetic covariance (r_A_), (2) the shared environmental covariance (r_C_), (3) the unique environmental covariance (r_E_), (4) the causal effect of trait X on trait Y (b_yx_), and (5) the causal effect of trait Y on trait X (b_yx_). Due to identification constraints, only three of the five potential sources of covariance (*b*_*yx*_, *b*_*xy*_, *r*_*A*_, *r*_*C*_, and *r*_*E*_) can be estimated in any model (Heath et al. 1993; Neale and Cardon 1992). As the data come from twins, the model also makes the standard assumptions of random mating, the absence of non-additive genetic variation, and gene-environment independence (Neale and Cardon 1992). Differences in the patterns of the cross-twin, cross-trait covariances enable testing causal hypotheses.

The likelihood ratio test can be used to compare the model fit of the DoC and models in which the DoC model is nested. E.g., the unidirectional DoC models are nested within the standard bivariate ACE model in which the 2 × 2 genetic and environmental covariance matrices are estimated. Hence, the likelihood ratio testing is appropriate (subject to regularity conditions; Steiger et al. 1985; Verhulst et al. 2019). If models are not nested, such as the bidirectional DoC model with additive genetic correlation (*r*_*A*_ estimated, *r*_*C*_ and *r*_*E*_ fixed) versus one with unshared environment correlation (*r*_*E*_ estimated and *r*_*A*_ and *r*_*C*_ fixed), a parsimony-based fit index such as Akaike Information Criterion (AIC) can be used. The AIC, calculated as twice the negative log-likelihood minus twice the degrees of freedom, balances model complexity and model fit to achieve a parsimony-related statistic. The model with the lowest AIC is the model of choice, in principle. Although the difference in log-likelihood between models with different numbers of mixture components may not meet the Steiger et al. (1985) regularity conditions, and hence may not be distributed as chi-squared, it is still useful as a guide to relative model fit.

### Structural Equation Mixture Modeling (SEMM)

Here we formally define the finite mixture distribution model. Let **y** denote a column vector of continuous observations, and *C* denote the number of mixture classes. The prior probability of belonging to latent class *i, ω*_*i*_, is equal to the proportion of the population belonging to that latent class. In a mixture model, the probability density function with C latent classes can be expressed as:

1 $$f\left({\varvec{y}}\right)=\sum_{i=1}^{C}{\omega }_{i}{\Phi }_{i}(y;{\theta }_{i})$$

where $\Phi$_*i*_ is the *i-th* density function, ${\theta }_{i}$ is the vector of the parameters of the *i-th* density function, and, as, above, *ω*_*i*_ is the corresponding mixture proportions, where $\sum {\omega }_{i}=1$. In the DoC mixture model, the class-specific densities $\Phi$_*i*_ $(y;{\theta }_{i})$ come from the same parametric family (multivariate normal) with class-specific mean and covariance matrices, which are a function of the ${\theta }_{i}$ parameter estimates. Combining structural equation modeling (SEM) with finite mixture modeling, known as Structural Equation Mixture Modeling (SEMM), involves specifying an SEM structure within each class (Dolan and van der Maas 1998; Lubke and Muthén, 2005; Vermunt and Magidson 2014; Yung 1997). The SEM structures must differ in some way to ensure the identification of the mixture proportions. This mixture modeling approach allows for the estimation of: (i) the number of classes, (ii) the parameters of the densities and the mixture proportions, (iii) each individual’s conditional class membership (posterior) probabilities, and (iv) the inference of the number of classes based on the estimation and testing of the mixture proportions.

### Direction of Causation twin mixture model (mixDoC)

**Fig. 2:**
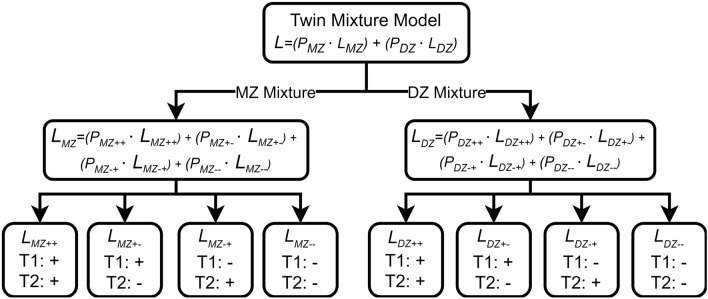
Schematic Diagram of the Mixture Direction of Causation (mixDoC) Twin Model. Figure illustrates the structure of the mixDoC model used to analyze the causal relationships in twin data. At the top level, the twin pairs are divided into two groups based on their zygosity: monozygotic (MZ) and dizygotic (DZ). The mixing proportions are estimated separately for each group. Within both the MZ and DZ groups, the model accounts for whether twins are concordant or discordant in their causal relationship between two traits, X and Y. Positive signs ( +) indicate that the causal direction is from X to Y, while negative signs ( −) indicate that the causal direction is from Y to X. The likelihood for each group (MZ and DZ) is calculated as the weighted sum of the likelihoods across the four possible combinations of concordant and discordant causal directions: concordant for X to Y in both twins (MZ +  + , DZ + +), concordant for Y to X in both twins (MZ–, DZ–), or discordant where one twin has X causing Y and the other twin has Y causing X (MZ + −, MZ− + , DZ + −, DZ− +). The weights (P) represent the estimated probabilities of twin pairs falling into these causal classes within each group

The model developed here is a mixture distribution DoC twin model (mixDoC), which considers two causal scenarios: one where X causes Y, and another where Y causes X. Since the DoC model uses pairs of twins as the sampling unit and each twin could be drawn from either causal distribution, there are four possible combinations for each family: two combinations where the twins are concordant for causal direction and two combinations where they are discordant. This gives rise to a constrained four mixture component twin model. Specifically: (1) X causes Y in both twins; (2) Y causes X in both twins; (3) X causes Y in twin 1 and Y causes X in twin 2; or (4) Y causes X in twin 1 and X causes Y in twin 2. Since the ordering of twins within a twin pair is random or unsystematic, the expected parameter estimates and mixing proportions of the discordant classes are expected to be equal. Figure 2 shows a schematic of the model (MZ and DZ twin pairs). For each group, four mixture component classes are modeled, with the mixing proportions estimated separately for MZ and DZ twin pairs. This means we estimate the proportions of concordance and discordance in causal direction specifically for each zygosity group, allowing for differences in the distribution of mixing proportion across zygosity.

The ability to resolve mixtures of opposing causal processes stems, in part, from differences in the observed means that arise from each causal process. To identify the DoC model within each concordant class, means for each trait (X and Y) are equated across twin 1 and twin 2 and across zygosities. In the discordant classes, because the causal process differs between the twins, the expected mean for each variable are different (µ_x1_ ≠ µ_x2_). However, the means in for each twin in the discordant classes correspond with the means from one of the other concordant class. Accordingly, while two means for each trait are estimated in the discordant classes, this does not increase the number of estimated parameters in the full mixDoC model. Phenotypic mean differences refer to the average differences in the same trait between different classes. In this study, this means the difference in the average levels of a trait (e.g., depression) between subpopulations with different causal directions (e.g., X causes Y versus Y causes X). For model identification, at least two of the parameters, *b*_*xy*_, *b*_*yx*_, *r*_*A*_, *r*_*C*_, or *r*_*E*,_ must be fixed to a specific value. As we focus here on the causal paths, two of the three parameters accounting for confounding (*r*_*A*_, *r*_*C*_, or *r*_*E*_) are fixed to zero. Constraining all three parameters to equal zero implies that there is no latent confounding due to genetic, shared environmental, or unique environmental sources. In that case, the only source of phenotypic covariance is the causal relationship. To limit the set of data-generating models, we considered two models: one with all background confounding set to zero (i.e., *r*_*A*_ = *r*_*C*_ = *r*_*E*_ = *0*) for all classes and unidirectional causation, and an extended model where the mixture still focused on unidirectional causation but also allowed for background genetic confounding (r_A_ was freely estimated but equated across classes).

This assumption—that r_A_ is the same across classes—implies that the genetic confounding effect does not differ between the causal processes (i.e., between the ‘X causes Y’ and ‘Y causes X’ classes). While this simplifies the model and simulation, it is a strong assumption. It is possible that the genetic confounding could vary between classes, reflecting different genetic influences on the causal pathways. As shown in Supplemental Fig. 1, this would result in four unique estimates for r_A_, one for the concordant for ‘X causes Y’, one for the concordant for ‘Y causes X’, and two for the cross-twin cross-trait covariance for the discordant class. Although we assume r_A_ is equated across classes in the main analysis, it is possible to estimate genetic confounding separately for each class as shown in Supplemental Table 1.

### Simulation design

All analyses were performed using R version 4.2.1 (R Core Team, 2021) with models fitted using OpenMx v2.20.6 (Neale et al. 2016). Code is available in a Github repository (https://github.com/Pvinh147/mixDoC). All data were simulated under a bivariate Gaussian mixture model, in which the data are generated from a finite mixture of bivariate Gaussian distributions.

For each simulation, eight separate datasets (four classes for each zygosity group) were generated and merged into MZ and DZ twin datasets. An overview of the simulation designs is provided in Table 1. Simulations were conducted with varying mixing proportions of concordant and discordant twin pairs at fixed parameter values to model different levels of heterogeneity. A high level of heterogeneity corresponds to an equal mix of individuals across classes (i.e., a 50/50 split between Class 1 and Class 2), while a low level of heterogeneity reflects a more imbalanced distribution (i.e., 95/5 split). These values were chosen to explore the feasibility of this mixture model rather than to exhaustively explore the multidimensional space.

**Table 1:** Simulation designs: this table provides an overview of the scenarios modeled in the simulation studies and their corresponding parameter of interest

Scenario	Parameter range	Explanation
Mixing Proportion (*P*_*MZ*+,_* P*_*MZ-*_,* P*_*DZ*+,_* P*_*DZ-*_*)*	*P*_*MZ*+_: 0.10 to 0.60, *P*_*MZ-*_ = *1−P*_*MZ*+_ *P*_*DZ*+_: 0.10 to 0.60, *P*_*DZ-*_ = *1−P*_*DZ*+_	Investigate how varying the proportion of twins exhibiting causal direction from X to Y ( +) or from Y to X (−) in both MZ and DZ groups affects classification accuracy
Presence of Bidirectional Causation (*P*_*MZ−bi*_, *P*_*DZ−bi*_)	*P*_*MZ-bi*_*:* 0.10 to 0.20*, P*_*MZ-uni*_ = *1−P*_*MZ-bi*_ *P*_*DZ-bi*_*:* 0.10 to 0.20, *P*_*DZ−uni*_ = *1−P*_*DZ−bi*_	Assesses the impact of bidirectional causality where twins exhibit both directions of causality (X to Y and Y to X, bi) on model performance
Phenotypic Mean Difference ($\Delta \overline{X }$, $\Delta \overline{Y }$)	0 to 1.5	Evaluates how differences in trait means influence the model’s ability to classify individuals
Causal Effect Size (*b*_*xy*_, *b*_*yx*_)	0.1 to 0.8	Examines how varying effect sizes of causal relationships affect model accuracy
Trait Heritability (*A*_*x*_*, A*_*y*_)	0.1 to 0.8	Investigates the impact of heritability (proportion of variance explained by genetic factors) on the model’s classification performance
Genetic Confounding (*r*_*A*_)	0.1 to 0.3	Explores how genetic confounding (genetic covariance) influences classification accuracy

The mixing proportions are freely estimated for each zygosity group. Consequently, the proportion of MZ twins exhibiting X to Y causation ( +) or Y to X causation (−) may be equal to or different from the corresponding proportions in DZ twins. P_MZ−uni_ and P_DZ−uni_ represent the total proportion of monozygotic (MZ) and dizygotic (DZ) twins exhibiting unidirectional causality, regardless of direction

A measure of entropy was used to evaluate the accuracy with which we can probabilistically assign individuals to classes based on the results of fitting the mixDoC model. The relative entropy index (Ramaswamy et al. 1993) is expressed as:

2 $$Ent=1-\frac{1}{NlnC}\sum_{i=1}^{N}\sum_{j=1}^{C}(-{p}_{ij}ln{p}_{ij})$$

where *N* is the number of observations, *C* is the number of classes, *p*_*ij*_ is the estimated posterior probability for individual *i* in class *j*. Entropy values range from zero to one, where values closer to one indicate better classification, meaning that the posterior probabilities approach zero or one for the two classes. To evaluate the entropy of the mixture model, we used parameter estimates averaged from 1000 replications. Parameters that we varied include: (i) strength of the causal effects; (ii) class means; (iii) modes of inheritance; and (iv) presence of genetic confounding.

**Fig. 3:**
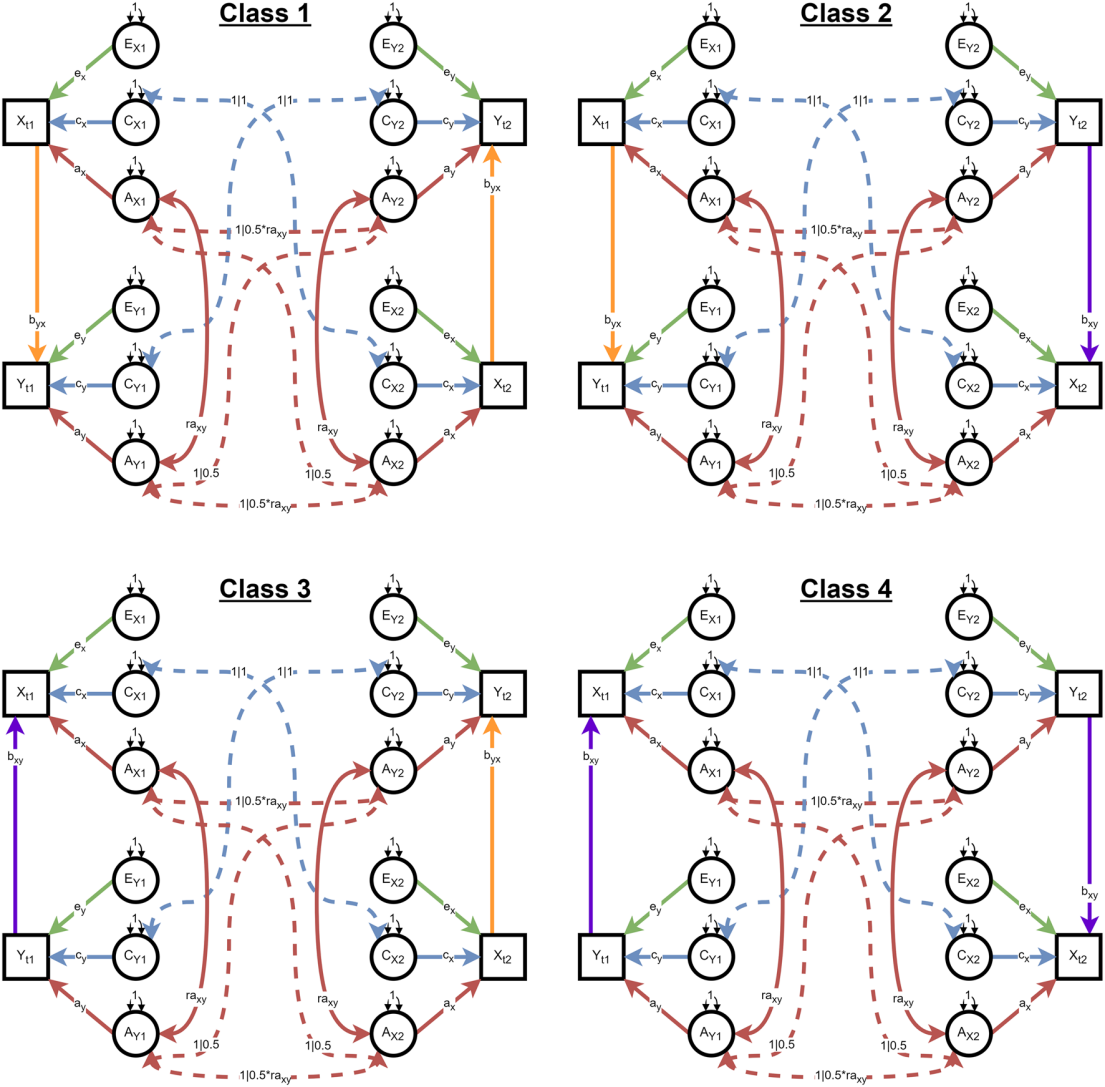
Path diagrams for the 4-class mixture Direction of Causation (mixDoC) twin model. Figure represents the four classes of causal relationships between traits X and Y within the 4-class mixDoC twin model. Each class (Class 1, Class 2, Class 3, and Class 4) depicts distinct directions of causality between the traits. In Class 1, twin pairs are concordant in the causal direction from X to Y (byx). Class 2 and Class 3 depict twin pairs discordant for causal direction. Class 4 depicts concordant causal direction from Y to X. Dashed lines represent covariances across twins

We fit the following models to each dataset: (i) the novel 4-class-per-zygosity mixture distribution (4-class), for which the path diagram is shown in Fig. 3; (ii) the concordant-pairs-only reduced mixture model (2-class); (iii) non-mixture X causes Y DoC; (iv) non-mixture Y causes X DoC; (v) non-mixture bidirectional DoC; and (vi) non-mixture bivariate ACE model with *r*_*A*_, *r*_*C*_, and *r*_*E*_ estimated, i.e., trait covariance is entirely due to the sharing of A, C, and E factors. The path diagrams for models ii-vi are provided in Supplemental Figs. 2, 3, 4, 5, 6.

## Results

### Model fit statistics

Model fit statistics for the mixture and non-mixture direction of causation twin models are presented in Table 2. The novel 4-class-per-zygosity mixDoC model and the competing models mentioned above were fitted to data with varying degrees of heterogeneity in the true data-generating model.

**Table 2:** Model fit statistics for Causal Models under Different Data Generating Scenarios: Table presents the model fit statistics for various causal models under different data-generating scenarios

A. Data Generating Model: Heterogenous—Unidirectional Causality (X → Y, Y → X), 30,000 Twin Pairs (5000 MZ and 5000 DZ Concordant for X → Y, 5000 MZ and 5000 DZ Discordant, 5000 MZ and 5000 DZ Concordant for Y → X)				
Model	df	−2LL	AIC	ΔAIC
4-Class mixture	119,984	144,293.4	144,325.4	
2-Class mixture	119,986	148,372.6	148,400.6	4075.2
DoC (X → Y)	119,991	148,864.1	148,882.1	4556.7
DoC (Y → X)	119,991	148,534.6	148,552.6	4227.2
Bidirectional	119,990	148,469.6	148,489.6	4164.2
Correlated factors	119,989	148,435.8	148,457.8	4132.4

B. Data Generating Model: Homogeneous—Bidirectional Causality, 10,000 Twin Pairs (5000 MZ and 5000 DZ exhibiting bidirectional relationship)				
Model	df	−2LL	AIC	ΔAIC
4-Class mixture	39,984	45,854.20	45,986.20	32.76
2-Class mixture	39,986	45,954.16	45,982.16	28.72
DoC (X → Y)	39,991	46,635.55	46,653.55	700.11
DoC (Y → X)	39,991	46,060.20	46,078.20	124.76
Bidirectional	39,990	45,933.44	45,953.44	
Correlated factors	39,989	45,933.44	45,955.44	2.0

C. Data Generating Model: Homogenous—Unidirectional Causality (X → Y), 10,000 Twin Pairs (5000 MZ and 5000 DZ Concordant for X→Y)				
Model	df	−2LL	AIC	ΔAIC
4-Class mixture	39,984	44,294.21	44,326.21	7.65
2-Class mixture	39,986	44,300.56	44,328.56	8.00
DoC (X → Y)	39,991	44,300.56	44,318.56	
DoC (Y → X)	39,991	44,427.32	44,445.32	126.76
Bidirectional	39,990	44,300.56	44,320.56	2.00
Correlated factors	39,989	44,300.56	44,322.56	4.00

The models include a 4-class mixture, 2-class mixture, unidirectional DoC (X → Y and Y → X), bidirectional, and correlated factors models. Fit statistics include degrees of freedom (df), minus twice the log-likelihood (−2LL), and Akaike Information Criterion (AIC)

### Equal proportions scenario

Table 2A compares the fit statistics across the DoC models under the condition where there are equal numbers of twin pairs exhibiting concordance from X to Y, Y to X, and discordance for the direction of causation (25% of each of the four mixture classes). The AIC of 144,325.4 for the data-generating model, the 4-Class mixture model, is the lowest, which is expected. The substantial difference in AIC, approximately 4000, indicates that with a sample size of 30,000 pairs, distinguishing between the mixture and non-mixture models yields informative results. This suggests that the mixDoC model can effectively capture the heterogeneity present in the data.

### Bidirectional causation scenario

Table 2B compares the fit statistics across the DoC models when the data-generating model is the bidirectional DoC model for all twins, where no mixture distribution is present. In this scenario, the mixDoC models show less parsimony compared to the bidirectional model. This difference is evident in the AIC column of Table 2B, where the AIC of 45,953.44 for the bidirectional model is lower (indicating better fit) than 45,955.44 for the correlated factors model and much lower than the 4-component mixture model AIC of 45,986.20. This suggests that in the absence of mixture distribution, simpler models may provide a better fit.

### Additional scenarios

Table 2C demonstrates the model fit statistics when data are simulated from a single distribution, specifically the X causes Y DoC model applied uniformly across all zygosity pairs. In this case, the mixDoC model shows less parsimony than the other models, as indicated by its higher AIC values. This suggests that, in the absence of heterogeneity, the mixDoC model does not offer an advantage over the simpler DoC models. Supplementary Table 1 reports results for additional scenarios with different proportions of concordant and discordant twin pairs. Overall, when data heterogeneity due to differing causal directions at the subpopulation level is present, the mixDoC models show greater parsimony, reflected by the lower AIC values.

### Entropy and classification accuracy

Table 3 presents entropy values for simulations assessing the impact on classification when varying the class-specific means, causal effect size, trait heritability, or genetic confounding. Entropy is a measure of classification accuracy, with higher values indicating better classification. In the context of the mixDoC model, higher entropy values suggest that the model is more likely to assign individuals to the correct causal class within zygosity (e.g., X causes Y vs. Y causes X).

**Table 3:** Entropy values for causal models under different simulation conditions

Phenotypic mean difference			Causal effect size		
$\Delta \overline{X }$	$\Delta \overline{Y }$	Entropy	b_yx_	b_xy_	Entropy
0.1	0.1	0.209	0.1	0.1	0.210
0.5	0.1	0.384	0.1	0.5	0.283
1.5	0.1	0.738	0.1	0.8	0.337
0.5	0.5	0.701	0.5	0.5	0.432
1.5	1.5	0.998	0.8	0.8	0.594

Additive genetic variance of trait Y		Genetic confounding	
*a*_*y*_	Entropy	*r*_*a*_	Entropy
0.1	0.286	0.1	0.260
0.3	0.238	0.15	0.249
0.4	0.233	0.20	0.238
0.5	0.231	0.25	0.237
0.6	0.230	0.3	0.230

Data were simulated for 5000 twin pairs with class proportions equal across classes and zygosity. Each section of the table varies only the specified parameter while all other parameters are held constant. For the section, Additive Genetic Variance of Trait Y, the additive genetic variance of X (a_x_) is fixed at 0.7

### Impact of phenotypic means

The results indicate that entropy depends heavily on the phenotypic means. Larger mean differences within the same trait across groups lead to higher entropy, reflecting better classification accuracy. For instance, if the mean of X (or/and the mean of Y) in the ‘X causes Y’ group is significantly higher than in the ‘Y causes X’ group, the model can more reliably classify individuals based on their trait levels. As shown in Table 3, increasing phenotypic mean differences results in higher entropy values, indicating improved classification accuracy.

This highlights the importance of phenotypic mean differences in the performance of the mixDoC model. Substantial phenotypic mean differences enable the model to accurately identify underlying causal structures, leading to precise estimates of causal effects and better classification of individuals.

### Impact of causal effect size

The results show that larger causal effect sizes improve classification accuracy. This is because stronger causal relationships create clearer distinctions between groups, making it easier for the model to classify individuals accurately. As indicated in Table 3, higher causal effect sizes correspond to increased entropy values, demonstrating better classification accuracy.

### Impact of trait heritability and genetic confounding

Classification accuracy deteriorates when the proportions of variance between the two traits become more similar. Additionally, the presence of covariance between the background A, C, or E factors for the two traits negatively impacts entropy, indicating that genetic and environmental confounding can obscure the causal relationships being modeled.

## Discussion

The model developed and tested in this article integrates the Direction of Causation (DoC) twin model with finite mixture modeling to address potential population heterogeneity due to differences in causal directions. Our results demonstrate that in the presence of data heterogeneity, mixture models exhibit better fit statistics, even with low levels of data heterogeneity. Conversely, the mixDoC twin model is less parsimonious when the population is homogeneous or when the causal effect is bidirectional. This finding underscores the importance of considering population heterogeneity in causal modeling, particularly in psychiatric and behavioral genetics where variations in causal direction are possible.

Analyses from our simulation studies show that the mixDoC model successfully recovers both parameter estimates and mixture proportions. Even with modest heterogeneity, the mixture model demonstrates greater parsimony than the unidirectional and bidirectional DoC models, underscoring its potential in scenarios where causal directions differ across subgroups. Additionally, this model detects latent heterogeneity and estimates individual conditional class membership probabilities, providing an insight into population heterogeneity. Although the model's performance is enhanced by larger phenotypic mean differences that are tied to the causal effects, it offers a promising framework for identifying heterogeneous causal relationships in twin data. In the main analysis, we constrained the additive genetic covariance (r_A_) to be equal across classes, simplifying the model. However, we also demonstrated that r_A_ can be freely estimated across classes, allowing for the possibility that genetic confounding varies between subpopulations, which may offer a more flexible approach in cases where genetic influences differ by causal direction.

As with all models, the mixDoC has limitations. Similar to the DoC twin model (Gillespie et al. 2003; Heath et al. 1993; Maes et al. 2021; McAdams et al. 2021; Neale and Cardon 1992; Verhulst and Estabrook 2012), it assumes random mating, no genotype-environment interactions, and no genotype-by-environment covariance. These assumptions simplify the model but may not hold true in all real-world scenarios. Both models are susceptible to measurement error, which may bias estimates of the causal effect if the amount of error differs substantially between traits. Measurement error is a critical issue in psychological and medical research, where precise measurement of constructs is challenging.

A prominent limitation of the mixDoC model, that is shared with other finite mixture models (Lubke and Muthén, 2005; McLachlan and Peel 2000; Nylund et al. 2007), is the need for large sample sizes to obtain accurate classification. This requirement arises because the model’s classification accuracy depends on large class separation, driven by phenotypic mean differences between classes and the magnitude of the causal effects. In the present case, the covariance structures from the different causal directions are not strongly distinct: they differ primarily in the across-variable across-relative correlations, which tend to be the smallest correlations observed. The presence of mean differences is very important for the detection of the mixture. This highlights a common trade-off in statistical modeling between model complexity and the power to detect effects.

A second limitation is the assumption that within each mixture class, the data are distributed as multivariate normal. Departures from multivariate normality, such as those induced by scaling artifacts, may lead to incorrect inferences. This is particularly important in behavioral research, where well-behaved data are often scarce and typically derived from questionnaires or direct observations. These departures could spuriously generate apparent evidence for a mixture distribution when none exists. Analyzing at the latent trait level could mitigate some of these issues. For domains such as neuroimaging or biochemical assays, where multivariate normality is more common, this assumption may be less problematic. However, in behavioral and psychological research, future work could explore alternative distributions or non-parametric approaches to address these limitations.

Several of these assumptions can be tested with additional data. For instance, the assumption of no A-C covariance can be tested by adding polygenic scores for the two traits to the model (Dolan et al. 2021). Random mating can be assessed by including marital pairs assessed on the same traits. Genotype-by-environment interactions may be tested at the observed level by including moderating variables (Purcell 2002), ideally exogenous causes of the traits to avoid collider bias. GxE interaction (Boker et al. 2023) at the latent level can be assessed when variables are measured continuously and multiple indicators of latent traits are available. Measurement error can be evaluated with test–retest protocols, ensuring an appropriate inter-test interval to reduce interference but avoiding true developmental change.

Although this method seems limited to pairs of relatives, it could, in principle, be used to assess the probability that a randomly selected individual or patient belongs to one of the mixture classes. Longitudinal data would be valuable in this context, with occasion 1 and occasion 2 serving as proxies for twin 1 and twin 2 to uncover latent population heterogeneity. A mixture distribution model for the cross-lagged panel design would be similar to the twin model presented here but would lack MZ and DZ groups, losing the ability to differentiate between individual-specific and familial sources of variation. However, the cross-lagged panel model could still offer valuable insights into the temporal dynamics of causal relationships.

Determining the causal direction at an individual level through calculating the posterior class membership probabilities may offer theoretical insights into variability in treatment response. In therapeutic settings, patients undergoing the same treatment often experience variability in outcomes, and one possible explanation could be differences in causal direction between comorbid conditions. Treating the condition that is causally downstream may provide temporary relief but could lack lasting effects if the upstream cause remains unaddressed. Conversely, addressing the upstream causal variable could, in theory, lead to improvements in both disorders. While this aligns with the goals of precision medicine, further empirical research is needed to assess the feasibility and practical applications of this approach in a clinical setting.

In conclusion, by integrating the Direction of Causation twin model with finite mixture modeling, we developed a model that accommodates heterogeneity due to subpopulations differing by causal direction. The mixDoC model has the potential to contribute towards explaining heterogeneity in treatment outcomes. While currently limited to multivariate normally distributed bivariate twin data, future developments could incorporate additional family members, multivariate, and longitudinal data. With multivariate data, specifying that the mixture distribution operates at the level of a latent factor with multiple indicators is possible. This approach could overcome the need for data to conform to the multivariate normal distribution within each mixture component class, making it more applicable to behavioral and psychological measures. Further research should explore these extensions and evaluate their performance in diverse empirical contexts.

## Supplementary Information

Below is the link to the electronic supplementary material.

Supplementary file1 (DOCX 1201 KB)

## Data Availability

R coding scripts for fitting the mixCLPM model can be found on GitHub at https://github.com/Pvinh147/mixDoC.
