# Supplementary material for: On the Detection of Population Heterogeneity in Causation Between Two Variables: Finite Mixture Modeling of Data Collected from Twin Pairs
Source: Behav Genet. 2024 Nov 26;55(1):59–70. doi: 10.1007/s10519-024-10207-9 (PMC11790744; doi:10.1007/s10519-024-10207-9)
Supplement: Supplementary file 1 — Supplementary file1 (DOCX 1201 KB) [file 10519_2024_10207_MOESM1_ESM.docx]

**Supplemental Materials**

**On the detection of population heterogeneity in causation between two variables: Finite mixture modeling of data collected from twin pairs.**

Corresponding Author

Philip B. Vinh, Virginia Institute for Psychiatric and Behavioral Genetics, Virginia Commonwealth University, Richmond VA 23298

Email: [vinhpb@vcu.edu](mailto:vinhpb@vcu.edu)
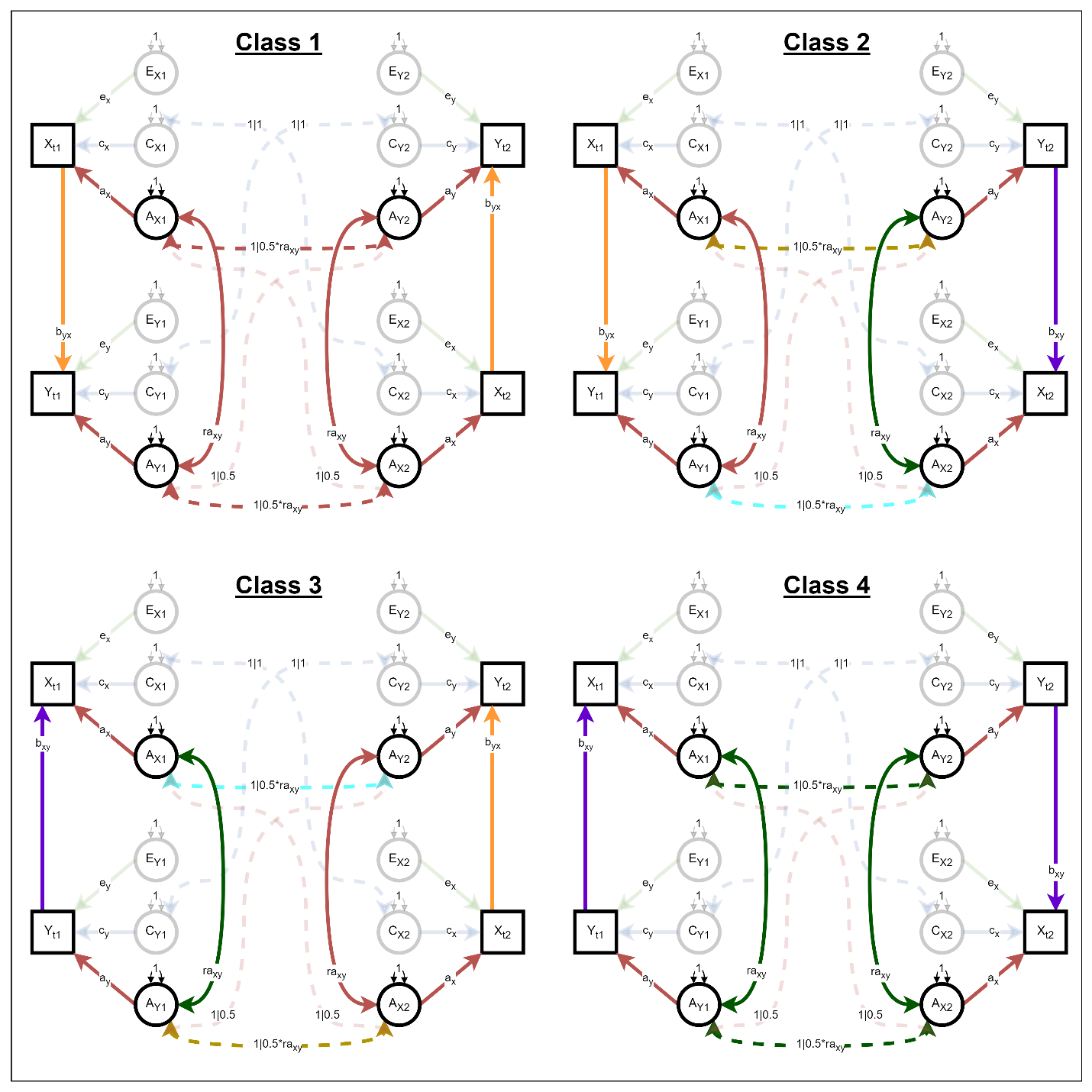


Supplementary Figure S1: Path diagrams for the mixture model illustrating the different genetic covariances (rA) between traits X and Y across four classes. Each class represents a distinct causal relationship, with the rA paths highlighted in different colors: maroon, gold, teal, and green. The maroon paths represent the genetic covariance for concordance in the causal direction from X to Y. The gold and teal paths from Class 2 and Class 3 represent the unique cross-twin cross-trait covariance. The green paths represent the genetic covariance for concordance in causal direction from Y to X. These paths illustrate how the genetic contributions to the covariance between X and Y vary across the different subpopulations within the model.

Supplementary Table S1: Parameter Recovery for When the genetic covariance is freely estimated between classes. The table displays the true parameter values alongside the estimated parameter values for two scenarios. Each simulation is of 20000 twin pairs at equal mixing proportions where the difference between simulations is due to differing trait mean separation.

| Cohen’s d | | X | 0.365 | | 0.716 | |
| --- | --- | --- | --- | --- | --- | --- |
|  |  | Y | 0.207 | | 0.804 | |
| Parameter | Simulated Value | | Estimate | SE | Estimate | SE |
| p_MZ+-_ | 0.25 | | 0.251 | 0.018 | 0.250 | 0.003 |
| p_MZ--_ | 0.25 | | 0.252 | 0.019 | 0.252 | 0.010 |
| p_DZ+__ | 0.25 | | 0.245 | 0.022 | 0.250 | 0.006 |
| p_DZ--_ | 0.25 | | 0.267 | 0.035 | 0.254 | 0.015 |
| b_12_ | 0.1 | | 0.094 | 0.026 | 0.100 | 0.006 |
| b_21_ | 0.4 | | 0.398 | 0.016 | 0.401 | 0.014 |
| a_11_ | 0.7 | | 0.700 | 0.006 | 0.700 | 0.006 |
| c_11_ | 0.2 | | 0.198 | 0.022 | 0.201 | 0.022 |
| e_11_ | 0.1 | | 0.100 | 0.001 | 0.100 | 0.001 |
| a_22_ | 0.3 | | 0.301 | 0.003 | 0.301 | 0.002 |
| c_22_ | 0.6 | | 0.600 | 0.004 | 0.600 | 0.004 |
| e_22_ | 0.1 | | 0.100 | 0.001 | 0.100 | 0.001 |
| ra_1_ | 0.1 | | 0.108 | 0.065 | 0.102 | 0.060 |
| ra_2_ | 0.2 | | 0.201 | 0.027 | 0.199 | 0.026 |
| ra_3_ | 0.15 | | 0.150 | 0.018 | 0.151 | 0.015 |
| ra_4_ | 0.12 | | 0.128 | 0.063 | 0.128 | 0.063 |


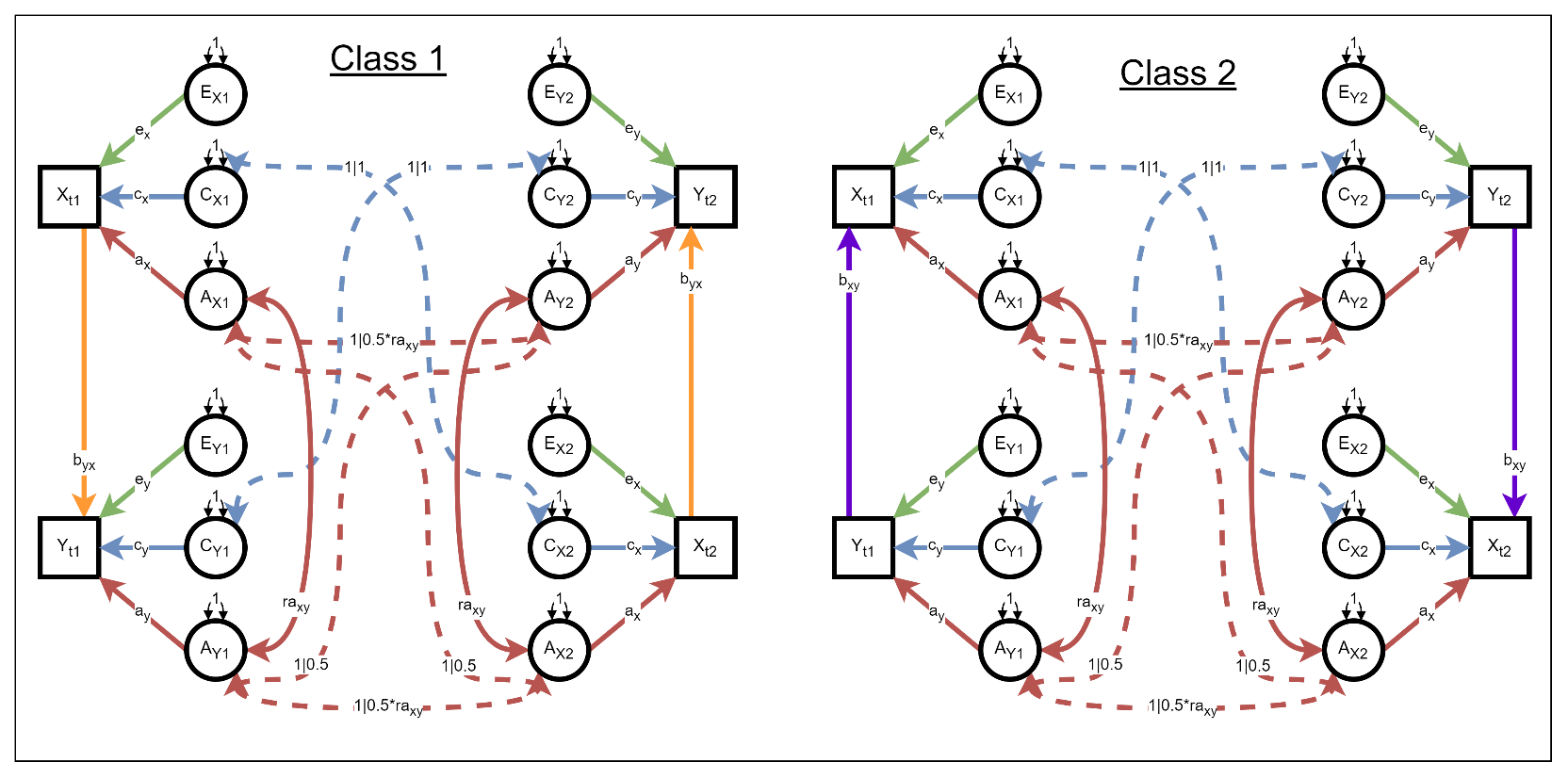


Supplementary Figure S2: Path diagram for the identified 2-class mixture model. This figure illustrates the path diagrams for the 2-class mixture model, with each class representing concordant causality among twin pairs. In Class 1, the causal direction is from X to Y (b_yx_), while in Class 2 the causal direction is from Y to X (b_xy_) Double-headed arrows represent the covariances between traits, while the dashed lines are added for visual clarity to show the cross-twin covariances.


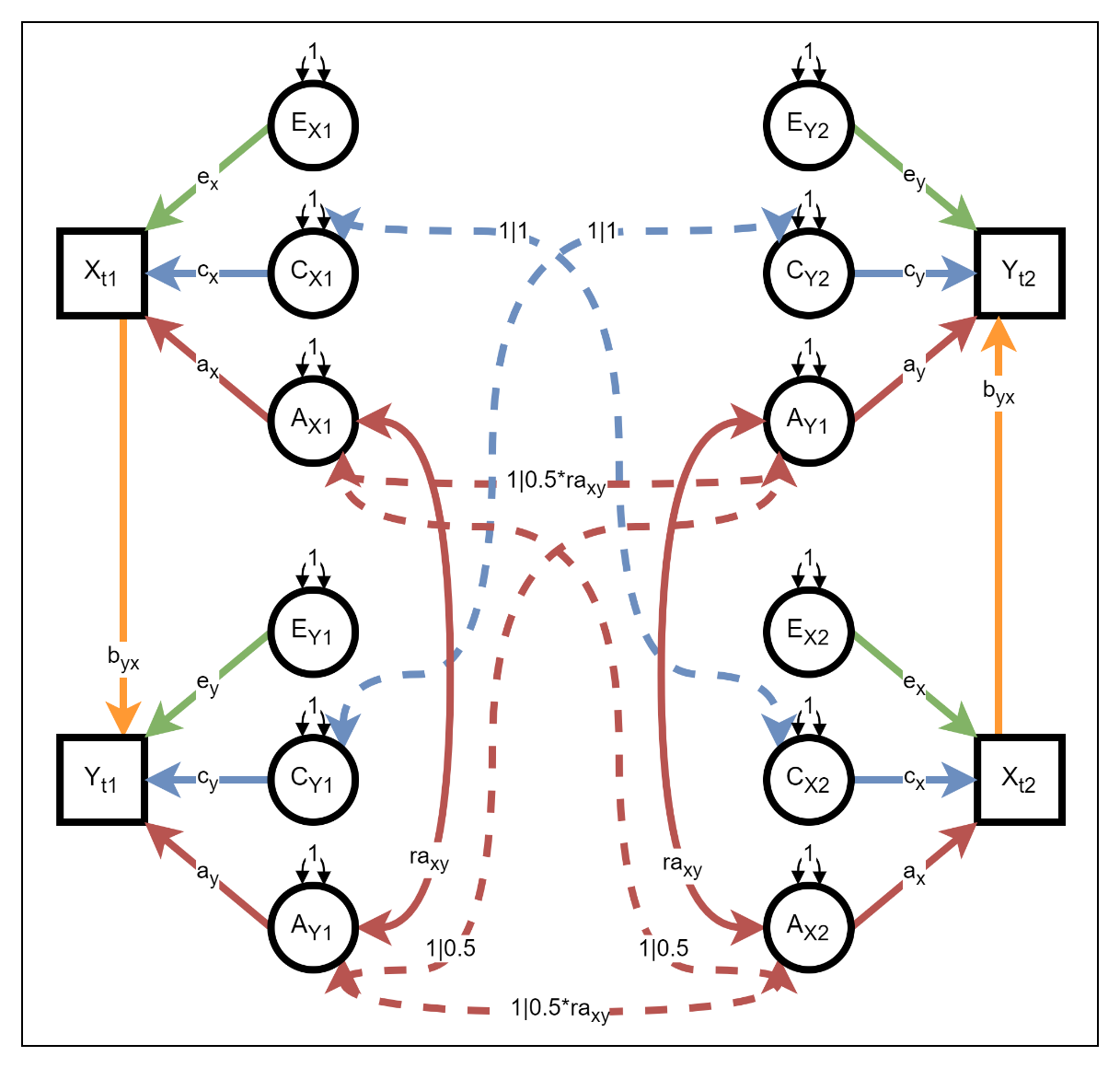


Supplementary Figure S3: Path diagram for the identified unidirectional Direction of Causation twin model (X to Y). This figure illustrates the path diagram for the non-mixture Direction of Causation twin model where the causal direction from X to Y (b_yx_) to estimated and the genetic confounding (r_a_). Double-headed arrows represent the covariances between traits, while the dashed lines are added for visual clarity to show the cross-twin covariances.


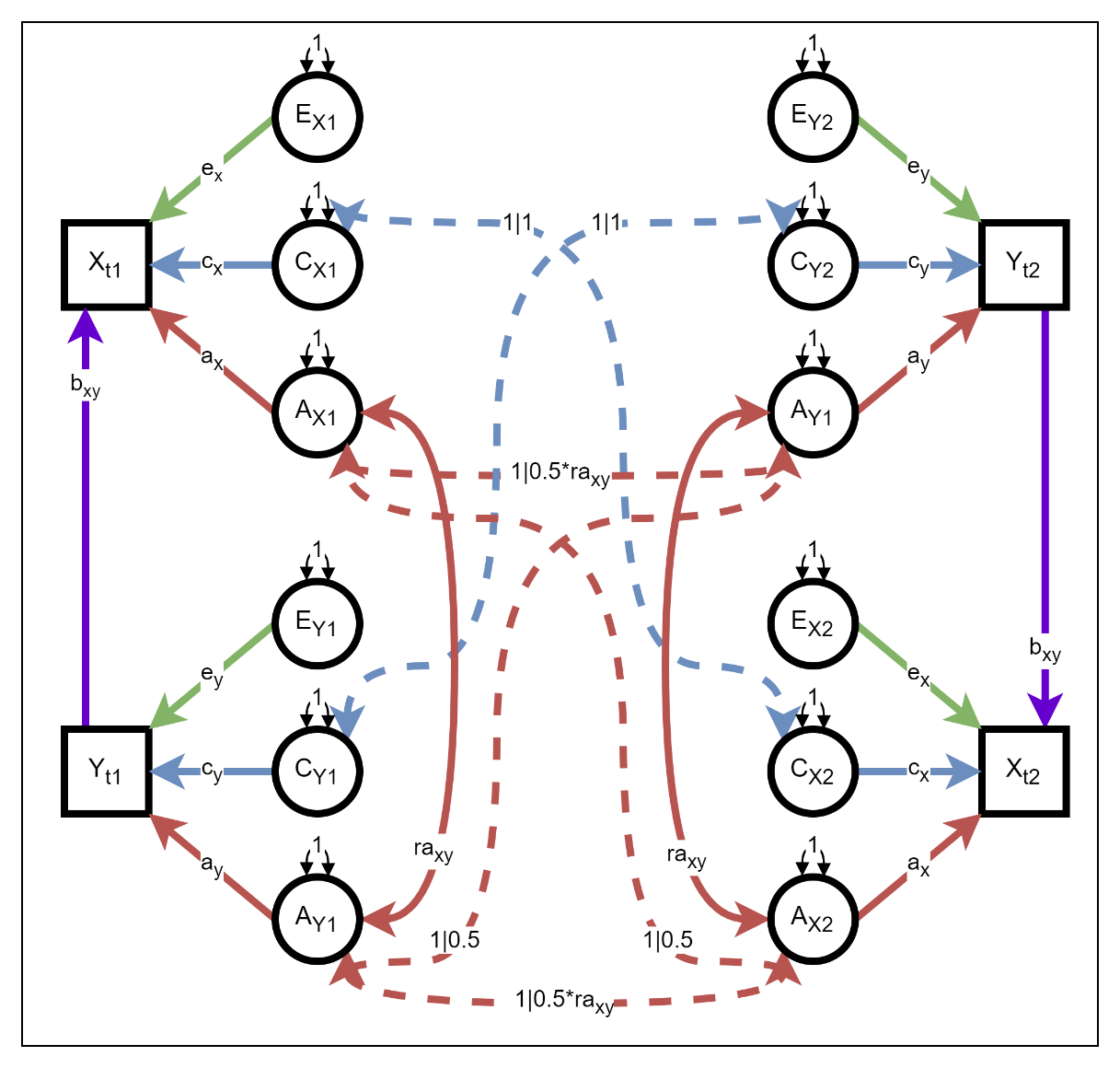


Supplementary Figure S4: Path diagram for the identified unidirectional Direction of Causation twin model (Y to X). This figure illustrates the path diagram for the non-mixture Direction of Causation twin model where the causal direction from Y to X (b_xy_) to estimated and the genetic confounding (ra). Double-headed arrows represent the covariances between traits, while the dashed lines are added for visual clarity to show the cross-twin covariances.


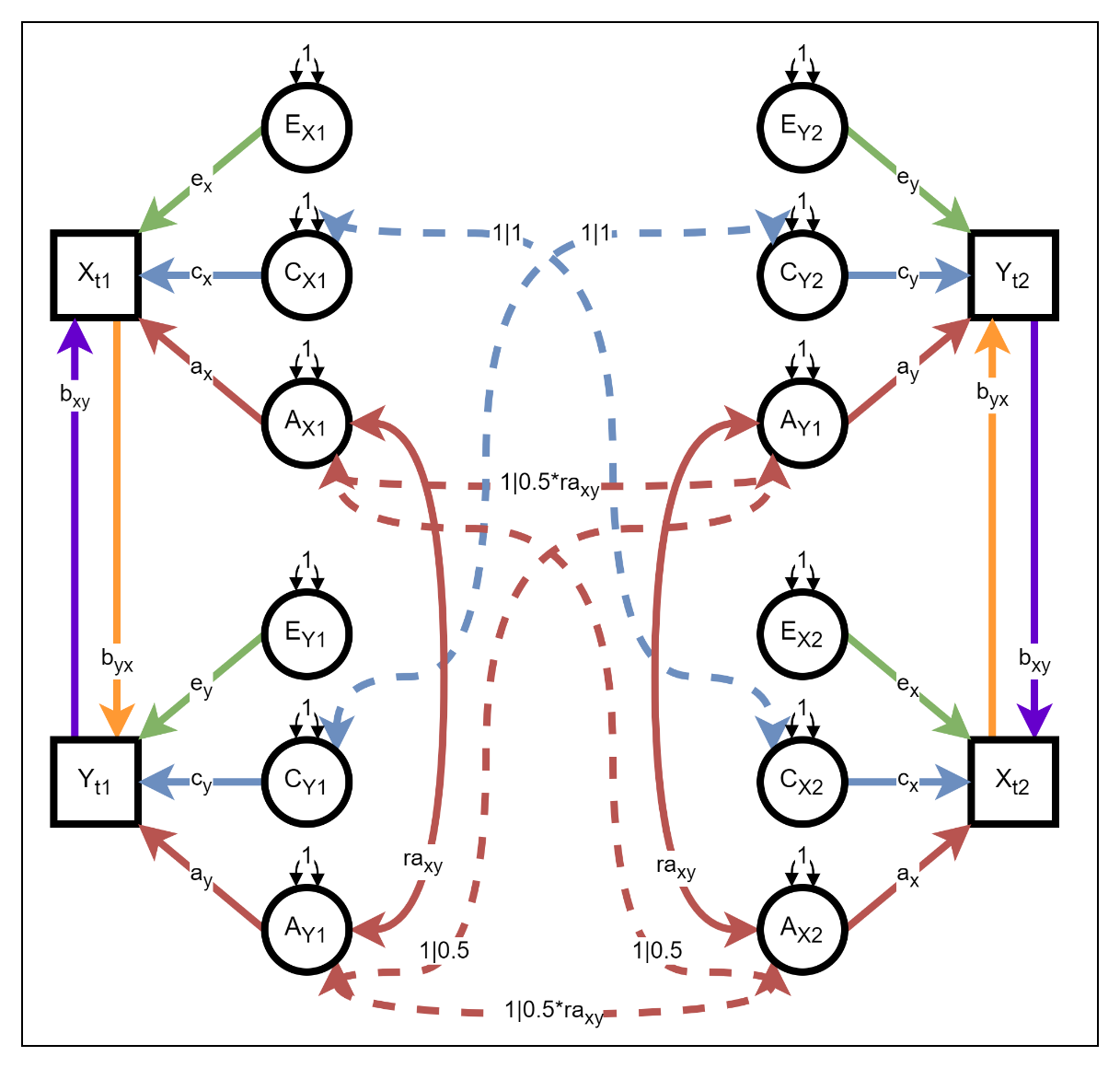


Supplementary Figure S5: Path diagram for the identified bidirectional Direction of Causation twin model (Y to X). This figure illustrates the path diagram for the Direction of Causation twin model the causal direction from Y to X, causal direction from X to Y, and genetic confounding are estimated. Double-headed arrows represent the covariances between traits, while the dashed lines are added for visual clarity to show the cross-twin covariances.


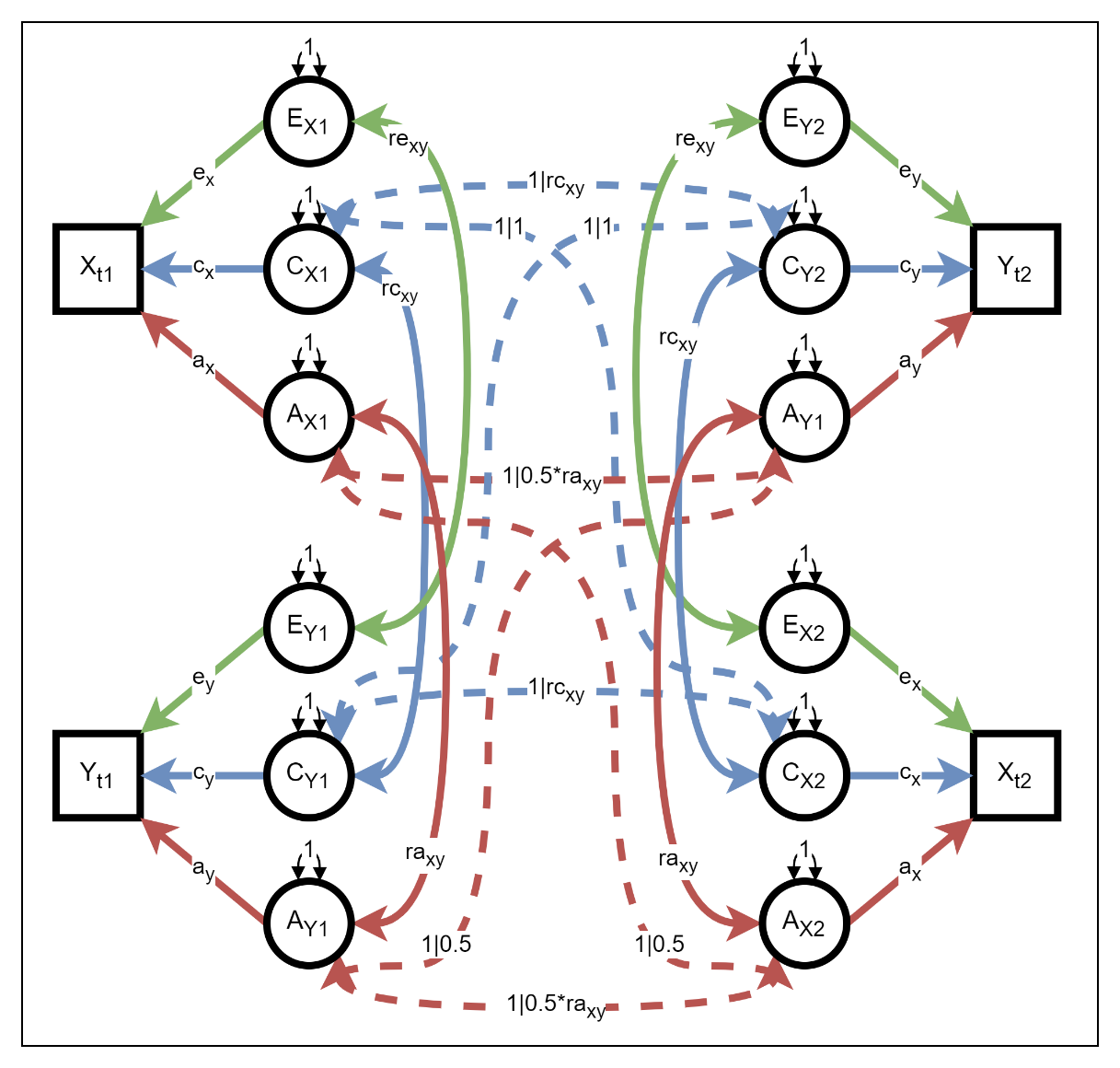


Supplementary Figure S2: Path diagram for the identified bivariate twin model. This figure illustrates the classical bivariate twin model used to estimate the genetic (A), shared environmental (C), and unique environmental (E) contributions to the covariance between two traits, X and Y. The genetic covariance (r_a_), shared environmental covariance (r_c_), and unique environmental covariance (r_e_) are estimated. Double-headed arrows represent the covariances between traits, while the dashed lines are added for visual clarity to show the cross-twin covariances.

**Supplemental Table S2.** Model Fit Statistics for Causal Models under Various Data Generating Scenarios

This table provides detailed model fit statistics for additional data-generating scenarios, exploring different levels of heterogeneity and sample sizes. The table includes degrees of freedom (df), minus twice the log-likelihood (-2LL), and Akaike Information Criterion (AIC) values for each scenario and model.

| Data Generating Model: | Homogenous - Unidirectional (Y → X),  10,000 Twin Pairs (5,000 MZ and 5,000 DZ Concordant for Y → X) | | | |
| --- | --- | --- | --- | --- |
| Model | df | -2LL | AIC | ΔAIC |
| 4-Class mixture | 39984 | 44295.95 | 44327.95 | 9.39 |
| 2-Class mixture | 39986 | 44295.84 | 44323.84 | 5.28 |
| DoC (X → Y) | 39991 | 45002.67 | 45020.67 | 702.11 |
| **DoC (Y → X)** | 39991 | 44300.56 | 44318.56 |  |
| Bidirectional | 39990 | 44300.56 | 44320.56 | 2.00 |
| Cholesky | 39989 | 44300.56 | 44322.56 | 4.00 |
| Data Generating Model: | Heterogeneous - Unidirectional (X → Y, Y → X),  20,000 Twin Pairs (5000 MZ and 5,000 DZ Concordant for X → Y, 5,000 MZ and 5,000 DZ Concordant for Y → X) | | | |
| Model | df | -2LL | AIC | ΔAIC |
| 4-Class mixture | 79984 | 89856.46 | 89888.46 | 4.77 |
| **2-Class mixture** | 79986 | 89855.69 | 89883.69 |  |
| DoC (X → Y) | 79991 | 90207.52 | 90225.52 | 341.83 |
| DoC (Y → X) | 79991 | 89955.85 | 89973.85 | 90.16 |
| Bidirectional | 79990 | 89888.51 | 89908.51 | 22.82 |
| Cholesky | 79989 | 89887.28 | 89909.28 | 25.59 |
| Data Generating Model: | Heterogeneous - Unidirectional (X → Y, Y → X),  10,000 Twin Pairs (4,800 MZ and 4,800 DZ Concordant for X → Y, 50 MZ and 50 DZ twin pairs Discordant, 50 MZ and 50 DZ Concordant for Y → X) | | | |
| Model | df | -2LL | AIC | ΔAIC |
| **4-Class mixture** | 40784 | 45366.06 | 45398.06 |  |
| 2-Class mixture | 40786 | 45392.89 | 45420.89 | 22.83 |
| DoC (X → Y) | 40791 | 45397.37 | 45415.37 | 17.31 |
| DoC (Y → X) | 40791 | 45522.79 | 45540.79 | 142.73 |
| Bidirectional | 40790 | 45397.31 | 45417.31 | 19.25 |
| Cholesky | 40789 | 45397.27 | 45419.27 | 21.15 |
| Data Generating Model: | Heterogeneous - Unidirectional (X → Y, Y → X) and Bidirectional,  10,000 Twin Pairs (2,500 MZ and 2,500 DZ Concordant for X → Y, 2,500 MZ and 2,500 DZ twin pairs Discordant, 2,500 MZ and 2,500 DZ Concordant for Y → X, 2,500 MZ and 2,500 DZ with Bidirectional Causation) | | | |
| Model | df | -2LL | AIC | ΔAIC |
| **4-Class mixture** | 40784 | 95960.57 | 95992.59 |  |
| 2-Class mixture | 40786 | 98179.51 | 98207.53 | 2214.94 |
| DoC (X → Y) | 40791 | 98179.51 | 98207.53 | 2214.94 |
| DoC (Y → X) | 40791 | 98179.51 | 98207.53 | 2214.94 |
| Bidirectional | 40790 | 98187.92 | 98207.93 | 2215.34 |
| Cholesky | 40789 | 98164.42 | 98186.43 | 2193.84 |
